# Screening of Potential Key Transcripts Involved in Planarian Regeneration and Analysis of Its Regeneration Patterns by PacBio Long-Read Sequencing

**DOI:** 10.3389/fgene.2020.00580

**Published:** 2020-06-16

**Authors:** Yibo Yang, Peizheng Wang, Baijie Jin, Zimei Dong, Guangwen Chen, Dezeng Liu

**Affiliations:** College of Life Science, Henan Normal University, Xinxiang, China

**Keywords:** *Dugesia japonica*, regeneration, full-length transcriptome sequencing, DETs, WGCNA

## Abstract

*Dugesia japonica* is an excellent animal model for studying the regeneration mechanism due to its characteristics of rapid regeneration and easy breeding. PacBio sequencing was performed on the intact planarians (In) and regenerating planarians of 1 day (1d), 3 days (3d), and 5 days (5d) after amputation. The aim of this study is to deeply profile the transcriptome of *D. japonica* and to evaluate its regenerate changes. Using robust statistical analysis, we identified 5931, 5115, and 4669 transcripts differentially expressed between 1d and In, 3d and In, 5d and In, respectively. A total of 63 key transcripts were screened from these DETs. These key transcripts enhance the expression in different regenerate stages respectively to regulate specific processes including signal transduction, mitosis, protein synthesis, transport and degradation, apoptosis, neural development, and energy cycling. Finally, according to the biological processes involved in these potential key transcripts, we propose a hypothesis of head regeneration model about *D. japonica*. In addition, the weighted gene co-expression network analysis provides a new way to screen key transcripts from large amounts of data. Together, these analyses identify a number of potential key regulators controlling proliferation, differentiation, apoptosis, and signal transduction. What’s more, this study provides a powerful data foundation for further research on planarians regeneration.

## Introduction

*Dugesia japonica*, a flatworm with strong regenerative capacity, lives in fresh water. *D. japonica* has become a hot topic in the research of regeneration mechanism due to its short regeneration cycle. However, so far, the regeneration mechanism is not very clear. At present, it is believed that the new cell source of planarian regeneration is neoblasts, which are the cellular basis of newborn tissues and organs (Wenemoser and Reddien, 2010). Meanwhile, apoptosis and autophagy provide energy for regeneration activities such as cell proliferation (Pellettieri et al., 2010). In addition, the position information regulates the differentiation of corresponding tissues at the correct position. For example, the Wnt regulates the anterior-posterior axis, Bmp regulates the dorsal-ventral axis, Wnt5 and Slit regulate the medial-lateral axis (Reddien, 2018). Finally, the size of the new and original tissues is adjusted so that their proportions are coordinated (Reddien and Sánchez Alvarado, 2004). Recently, the genome of the asexual *D. japonica* clonal strain was sequenced (An et al., 2018). This is a major advance in genomic research of *D. japonica*. It is regrettable that the reads acquired from second-generation sequencing were too short to assemble complex genomes. Despite trying the new assembly strategy, the obtained genome sequences were still fragmented (An et al., 2018). In addition, transcriptome sequencing of early regeneration with *D. japonica* reveals that heat shock protein and MAPK pathway are involved in early response of regeneration. A schematic model based on the regulation network among apoptosis, autophagy and related signaling pathways is proposed (Yuan et al., 2018).

Although transcriptome sequencing is widely used in the study of regeneration, the accuracy of the obtained sequences is not high due to the lack of a reliable reference genome for *D. japonica* and the incomplete transcriptome splicing obtained from the second-generation transcriptome sequencing. The full-length transcriptome sequencing based on PacBio SMRT single-molecule real-time sequencing technology does not require disruption of the RNA fragments. The ultra-long read (median 10 kb) of this platform contains a single complete transcript sequence information, which avoids assembly errors (Sharon et al., 2013). The full-length transcriptome sequencing is often used to construct the unigene library to obtain the reference sequences on transcriptome level, which provides a good genetic information basis in the absence of a reference genome (Gordon et al., 2015). In this study, we performed the full-length transcriptome sequencing on the intact and regenerating worms, and then combined with the data from the second-generation sequencing to ensure the accuracy of structure and sequence integrity as much as possible. The data set for the early and medium regeneration stages provided a more comprehensive dynamic gene expression network in controlling regeneration. Based on the above data, we have identified some potential key transcripts for each regeneration stage by pairwise comparisons and weighted gene co-expression network analysis (WGCNA). We proposed a regeneration model at different time points according to the function of these transcripts. This work provides an important data foundation focusing on molecular regulation underlying regeneration in planarians.

## Materials and Methods

### Planarian Growth and RNA Sample Collection

**FIGURE 1:**
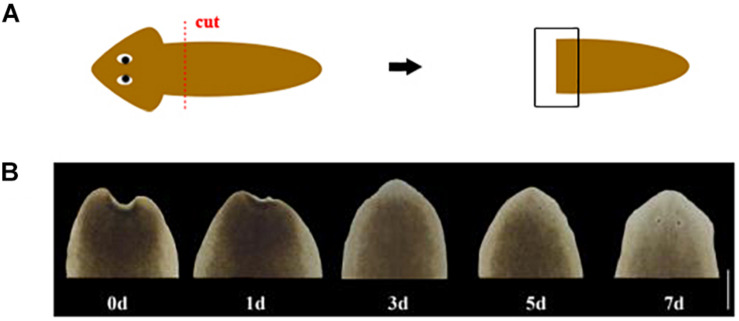
**(A)** Schematic of amputation. The red dotted line represents the cut position. The black box represents the area of regeneration for morphological observation. **(B)** Morphological changes during head regeneration (d: day after amputation. Scale bar: 0.5 mm).

Four different treatments were carried out on *D. japonica* (asexual strain) which was collected from Shilaogong (Hebi City, China) and then was asexually reproduced in laboratory (Dong et al., 2019). This study does not involve endangered or protected species, and the collection of specimen is approved by the Forestry Department of Wild Animal Protection, Henan Province, China. The sample points were set intact worms (named In), tail fragments at 1 day (named 1d), 3 days (named 3d), and 5 days (named 5d) after amputation, respectively. The worms about 1cm long were selected after a week of starvation at least. The cutting position of tail fragment is at pre-pharynx and post-auricle level (Figure 1). One worm or one fragment was used for each sample. After sampling, these tissues were quickly frozen in liquid nitrogen and stored at −80°C until RNA isolation. Three biological replicates were used for each of the sampling points.

### RNA Isolation, cDNA Library Construction, Transcriptome Sequencing, and Gene Expression Analysis

Total RNA was extracted from 12 samples using RNAiso plus reagent (Takara, Japan), and quality of RNA was assessed using the NanoDrop, Agilent 2100 Bioanalyzer and Electrophoresis. For third-generation transcriptome sequencing, equal mass of the total RNA from 12 samples were pooled together as the template for cDNA synthesis with the SMARTer PCR cDNA Synthesis Kit (Clontech, United States). 3 libraries were classified according to cDNA fragments size (1–2, 2–3, 3–6 kb) by using BluePippin Size Selection System. Finally, a total of six SMRT cells were sequenced on PacBio RS II. Subreads were obtained by filtering out the polymerase reads that the length is less than 50 bp, the accuracy is less than 0.75, or the sequence with joint. ROI (the reads of insert) sequences were extracted from the original sequences according to the condition that full passes ≥ 0 and the sequence accuracy was greater than 0.75. Consensus sequences of isoform were produced and corrected by Iterative isoform-clustering algorithm and Quiver software, and then the low-quality isoforms were corrected in aid of Illumina short reads using Proovread tool (Hackl et al., 2014; Gordon et al., 2015). All the downstream analyses were based on non-redundant sequences after removing redundant transcripts by CD-HIT (Li and Godzik, 2006).

For second-generation transcriptome sequencing, 12 libraries constructed from each sample (each sample has three biological replicates) were sequenced on Illumina HiSeq instrument. Clean data in the format of FASTQ was obtained after removing the reads contained adapter and low-quality reads from raw data. Function annotation and expression analysis of transcripts were carried out by BLAST and RSEM respectively (Altschul et al., 1997; Li and Dewey, 2011). Quantification of transcripts expression levels were estimated by fragments per kilobase of transcript per million mapped reads (FPKM). Principal component analysis (PCA) was performed using the ggbiplot R package.

### Differential Expression and Functional Enrichment Analysis

Tail fragments at 1, 3, and 5 days after amputation were sampled for test groups respectively, and the intact worms were sampled for the control group. Differential expression analysis was performed using the DESeq (Anders and Huber, 2010). Differentially expressed transcripts (DETs) were obtained with the condition of false discovery rate (FDR) < 0.01 and absolute fold change ≥ 2 in each pairwise comparison. Gene function was annotated based on the following databases: NR (NCBI non-redundant protein sequences); Pfam (Protein family); COG/eggNOG (Clusters of Orthologous Groups of proteins/evolutionary genealogy of genes: non-supervised orthologous groups); Swiss-Prot (A manually annotated and reviewed protein sequence database); KEGG (Kyoto Encyclopedia of Genes and Genomes); GO (Gene Ontology).

### Quantitative Real Time PCR Analysis (qRT-PCR)

To further validate the confidence of transcriptome sequencing data, six DETs were selected and analyzed via qRT-PCR. The qRT-PCR analysis was performed with three biological and three technical replicates. The RNA samples conformed to the required purity criteria (A260/A230 > 2.0, and A260/A280 of 1.8–2.0), and the integrity of the RNA samples was assessed by agarose gel electrophoresis. Then, 1 μg of total RNA was reverse transcribed into cDNA using the HiScript II Q RT SuperMix for qPCR (+gDNA wiper) (Vazyme, China). Quantitative real-time PCR was performed using AceQ qPCR SYBR Green Master Mix (Vazyme) according to the manufacturer’s instructions in 20 μl reactions. The PCR amplification procedure was carried out at 95°C for 300 s, followed by 40 cycles at 95°C for 10 s and 60°C for 30 s; this was followed by disassociation curve analysis in LightCycler^®^ 96 System (Roche). The *Elongation factor 2* (*Ef2*) gene was used as an internal reference. The comparative Ct method (2-△△Ct method) was used to calculate the relative gene expressions of the samples, which were normalized using the *DjEf2* mRNA level. Six transcripts were randomly selected to design primers based on the software PP 5.0, and all primers for this study are listed in Supplementary Table S1. The genes’ log2 fold change values of qRT-PCR and RNA-Seq were used for graphical presentation.

### Weighted Gene Co-expression Network Analysis (WGCNA)

To identify candidate genes and networks from regeneration DETs, weighted gene co-expression network analysis (WGCNA) was conducted to identify specific modules of co-expressed genes associated with each regeneration stage. Modules were defined as clusters of highly interconnected genes, and genes within the same cluster have high correlation coefficients among them. WGCNA was performed according to Langfelder and Horvath (2008). Prior to WGCNA, low-quality transcripts (the meanFPKM of all samples is less than 1) were filtered out to improve the accuracy of the resulting network. Transcripts can be clustered into different modules, which were classified and clustered by Dynamic Hybrid Tree Cut algorithm with minModuleSize is 30 and minimum height for merging modules is 0.12335. Module eigengenes were used to calculate correlation coefficients with samples. Weight refers to the connection strength between two transcripts in terms of the topology overlap measure. The weights across all connection strength of a transcript are summed and used to define the level of connectivity (Tai et al., 2018). The top 150 transcripts with high connectivity in each module were selected at first, and then the top 30 transcripts with high connection strength were screened from the 150 transcripts. Finally, the 30 transcripts considered hub genes. For transcripts in each module, KEGG pathway enrichment analyses were conducted to analyze the biological functions of modules.

## Results

### Morphological Observation During Head Regeneration

*D. japonica* in this study was cultured in a constant temperature incubator at 20°C and returned to normal form within 5 days following amputation. The wound can quickly shrink after cutting, and then the appearance of a transparent film can be observed on the 1st day of regeneration. On the 3rd day of regeneration, the white blastema grew and the length was between 0.1 and 0.25 mm. The regenerated eye spots can be observed in some worms. On the 5th day of regeneration, the eye spots of all worms appeared. The triangular head began to take shape, and the blastema began to have pigmentation. The auricle appeared on the 7th day of regeneration and the heads of most worms are lighter in color (Figure 1).

### Transcriptome Sequencing Analysis on Planarians

The morphological characteristics and organization functions of planarians were basically restored on the 5th day of regeneration. In order to further explore the molecular regulation underlying these morphological changes, transcriptome sequencing on these regenerative and intact planarians was performed. Three libraries mixed all samples were conducted for SMRT sequencing and acquired 16.17 Gb clean data. A total of 441945 ROI (The reads of insert) sequences were extracted from original sequences. Twelve libraries contained each sample respectively were conducted for RNA-Seq and 102.35 Gb clean data was obtained in total. Finally, 44655 non-redundant sequences for structural analysis and functional annotation were obtained (Table 1). Based on these non-redundant sequences of each sample, 31278 transcripts were annotated to eight databases (Table 2). PCA revealed that the 12 samples could be clearly assigned to four groups as In, 1d, 3d, 5d (Supplementary Figure S1), suggesting that the overall thranscriptome profiling was similar for three repetitions.

**TABLE 1:** Numbers of sequencing results.

**Sample**	**cDNA size**	**Reads of insert**	**Number of full-length non-chimeric reads**	**Non-redundant sequences**
F01	All	441945	230585	44655

**TABLE 2:** Numbers of annotated transcripts.

**Database**	**All**	**GO**	**KEGG**	**Pfam**	**Swissprot**	**COG**	**eggNOG**	**nr**
Annotated_Number	31278	7702	13434	18743	17328	9857	27346	30553

### Identification of DETs Among Different Regeneration Stages

To identify DETs at different time points of regeneration, we performed a comparison between the different regenerate time groups and the intact worms group respectively, and a great number of DETs were obtained with the condition of FDR < 0.01 and absolute fold change ≥ 2 in each pairwise comparison (Supplementary Table S2). At the group of 1d, a total of 5066 DETs were annotated into each database, of which 2558 DETs were annotated into the KEGG database, with a total of 251 pathways involved. In the 3d group, a total of 4432 DETs were annotated into each database. Among them, there were 2182 DETs annotated to the KEGG database, and a total of 238 pathways were involved. In the 5d group, a total of 4057 DETs were annotated into each database. Among them, there were 2058 DETs annotated to the KEGG database, involving 228 pathways in total (Supplementary Table S3). The number of DETs in group 1d is the largest. qRT-PCR analysis was used to validate the quality of transcriptome sequencing. Six DETs from each regenerated stage were selected for qRT-PCR. The one-to-one correspondence between the qRT-PCR and RNA-Seq data indicated the reliability of transcriptome data (Supplementary Figure S2).

Based on the number of transcripts involved in pathway (*P* < 0.01), the top six pathways were focused on (Supplementary Table S4). We found that many different transcripts were encoded as the same protein in these pathways. Then we selected the transcripts predicted to be the same protein and further screened transcripts with high expression levels and high differential expression folds (FPKM > 10, absolute log2 fold change ≥ 2).

In the first six pathways of the 1d up-regulation group, 16 transcripts encoded seven proteins respectively were screened. These proteins are heat shock 70 kDa protein 1/2/6/8 (HSPA1s), mitogen-activated protein kinase kinase kinase 3 (MAP3K3), phosphoenolpyruvate carboxykinase (PCK), polo-like kinase 4 (PLK4), beta-galactosidase (GLB1), V-type H+-transporting ATPase subunit a (ATPeV0A), and actinin alpha 1/4 (ACTN1_4) (Table 3). While 32 transcripts were screened in the first six pathways of the down-regulation group at 1d. These transcripts encoded six proteins respectively, including collagen COL1A and COL4A, tubulin TUBA and TUBB, adenylate cyclase 9 (ADCY9), and dihydropyrimidinase (DPYS) (Supplementary Table S5). Among the first six pathways in the 3d up-regulation group, 14 transcripts encoded eight proteins respectively were screened. These proteins are GLB1, ATPeV0A, clathrin heavy chain (CLTC), 6-phosphofructokinase 1 (PFK), PCK, ATP citrate (pro-S)-lyase (ACLY), Long-chain-fatty-acid–CoA ligase ACSBG and carnitine O-palmitoyltransferase 1 (CPT1A) (Table 4). Among the first six pathways of the 3d down-regulation group, 32 transcripts encoded six proteins respectively were screened. These proteins are ADCY9, Pyruvate kinase (PK), PFK, COL1A, TUBA, and TUBB (Supplementary Table S6).

**TABLE 3:** FPKMs and KEGG pathways of representative transcripts which are significantly up-regulated in 1d.

**KEGG**	**Pathway**	**Definition**	**ID**	**In (FPKM)**	**1d (FPKM)**
K03283 HSPA1s	MAPK signaling pathway	Heat shock 70 kDa protein 1/2/6/8	F01.PB6917	119.94	880.39
			F01.PB7357	113.10	492.96
			F01.PB17197	25.31	481.15
			F01.PB33477	1.13	26.55
K04421 MAP3K3, MEKK3	MAPK signaling pathway	Mitogen-activated protein kinase kinase kinase 3	F01.PB16966	0.03	14.46
K01596 E4.1.1.32, pckA, PCK	FoxO signaling pathway; Pyruvate metabolism; Citrate cycle (TCA cycle)	Phosphoenolpyruvate carboxykinase (GTP)	F01.PB30594	0	607.66
			F01.PB13715	15.08	222.02
			F01.PB44132	1.90	34.46
			F01.PB23564	1.04	17.23
			F01.PB13344	0.36	10.0
K08863 PLK4	FoxO signaling pathway	Polo-like kinase 4	F01.PB27496	2.48	231.61
			F01.PB1007	0.36	141.73
			F01.PB11041	5.49	27.25
K12309 GLB1, ELNR1	Lysosome	Beta-galactosidase	F01.PB32974	0.44	10.44
K02154 ATPeV0A, ATP6N	Lysosome	V-type H+-transporting ATPase subunit a	F01.PB2723	1.08	11.00
K05699 ACTN1_4	Focal adhesion	actinin alpha 1/4	F01.PB2671	1.41	61.04

**FPKM in this table means the meanFPKM of three replicates. The below tables are same.**

**TABLE 4:** FPKMs and KEGG pathways of representative transcripts which are significantly up-regulated in 3d.

**KEGG**	**Pathway**	**Definition**	**ID**	**In (FPKM)**	**3d (FPKM)**
K12309 GLB1, ELNR1	Lysosome	Beta-galactosidase	F01.PB32974	0.44	11.53
K02154 ATPeV0A, ATP6N	Lysosome	V-type H+-transporting ATPase subunit a	F01.PB14670	6.67	31.57
			F01.PB2723	1.08	10.23
K04646 CLTC	Lysosome	Clathrin heavy chain	F01.PB21704	2.64	64.62
K00850 pfkA, PFK	Glycolysis/Gluconeogenesis; Galactose metabolism	6-phosphofructokinase 1	F01.PB30251	1.79	15.67
K01596 E4.1.1.32, pckA, PCK	Glycolysis/Gluconeogenesis; Citrate cycle (TCA cycle)	Phosphoenolpyruvate carboxykinase (GTP)	F01.PB13715	15.08	229.30
			F01.PB15415	93.62	497.51
			F01.PB23564	1.04	10.9
			F01.PB30594	0	707.91
			F01.PB44132	1.90	15.78
K01648 ACLY	Citrate cycle (TCA cycle)	ATP citrate (pro-S)-lyase	F01.PB40974	0	10.71
K15013 ACSBG	Fatty acid degradation; fatty acid metabolism	Long-chain-fatty-acid–CoA ligase ACSBG	F01.PB17057	27.34	200.064
K08765 CPT1A	Fatty acid degradation; fatty acid metabolism	Carnitine O-palmitoyltransferase 1, liver isoform	F01.PB12384	1.43	43.81
			F01.PB2659	0.08	11.38

In the first six pathways of the 5d up-regulation group, 19 transcripts encoded seven proteins respectively were screened. These proteins are translation initiation factor 4G (EIF4G), cytoplasmic FMR1 interacting protein (CYFIP), tumor necrosis factor (TNF) receptor-associated factor 2 (TRAF2), HSPA1s, PFK, PCK, and COL1A (Table 5). While 37 transcripts were screened in the first six pathways of the down-regulation group at 5d. These transcripts encoded seven proteins respectively, including ADCY9, TUBA, TUBB, PFK, ATP-binding cassette ABCC1, and ABCA3 as well as COL1A (Supplementary Table S7).

**TABLE 5:** FPKMs and KEGG pathways of representative transcripts which are significantly up-regulated in 5d.

**KEGG**	**Pathway**	**Definition**	**ID**	**In (FPKM)**	**5d (FPKM)**
K03260 EIF4G	RNA transport	Translation initiation factor 4G	F01.PB40614	2.69	11.21
K05749 CYFIP	RNA transport	Cytoplasmic FMR1 interacting protein	F01.PB17559	3.98	22.72
K03173 TRAF2	MAPK signaling pathway	TNF receptor-associated factor 2	F01.PB18474	2.84	12.18
			F01.PB10263	0.41	12.33
K03283 HSPA1s	MAPK signaling pathway	Heat shock 70 kDa protein 1/2/6/8	F01.PB17197	25.31	533.55
			F01.PB7357	113.10	457.94
			F01.PB33477	1.13	13.27
K00850 pfkA, PFK	RNA degradation	6-phosphofructokinase 1	F01.PB30251	1.79	28.34
K01596 E4.1.1.32, pckA, PCK	Pyruvate metabolism	Phosphoenolpyruvate carboxykinase (GTP)	F01.PB13715	15.08	269.84
			F01.PB23564	1.04	10.44
			F01.PB30594	0	516.5
			F01.PB44132	1.90	10.9
			F01.PB7097	6.53	42.23
K06236 COL1A	ECM-receptor interaction	Collagen, type I, alpha	F01.PB14701	0.27	15.58
			F01.PB20259	0.74	16.74
			F01.PB31198	0.04	26.9
			F01.PB37046	5.20333	23.05
			F01.PB7606	0.07	10.89
			F01.PB9634	0.10	13.93

The encoded proteins of these DETs were involved in one or several different KEGG pathways and will be the focus of our future research. Among these representative proteins, PCK, HSPA1s, GLB1, and ATPeV0A played an important role in the entire regeneration process. COL1A, TUBA, TUBB, and ADCY9 were down-regulated throughout the regeneration process. In addition, some proteins were complexly regulated during regeneration. For example, there were both up-regulated and down-regulated transcripts encoding PFK to jointly regulate the function of the protein.

### Construction of Gene Co-expression Network

**FIGURE 2:**
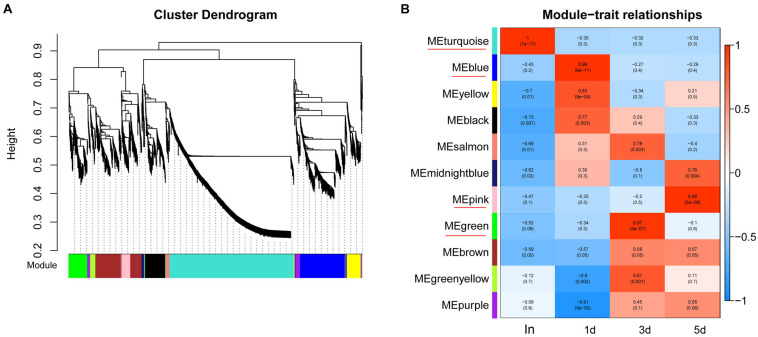
WGCNA of DETs at each time points of regeneration. **(A)** Hierarchical cluster tree showing co-expression modules identified by WGCNA. Each leaf in the tree represents one gene. The major tree branches constitute 11 modules which labeled with different colors. **(B)** Module–sample relationships. Each row corresponds to a module, labeled with a color as in **(A)**. Each column corresponds to a specific regeneration stage. The color of each cell at the row-column intersection indicates the correlation coefficient between the module and the regeneration stage. The module have the highest degree of correlation with a specific sample is indicated with red underline.

To obtain a comprehensive understanding of genes and to identify the specific genes expressed in the different regeneration stages, WGCNA on DETs was performed. WGCNA revealed that the transcripts can be clustered into 11 modules (labeled with different colors) (Figure 2A and Supplementary Table S8). Four out of 11 co-expression modules were selected that have the highest degree of correlation with one of the samples respectively. The four modules were indicated with red underlines in Figure 2B. The turquoise module identified 1442 transcripts specific to the intact group (In). The blue module identified 537 transcripts specific to the 1d group. The green module, representing 216 transcripts, was highly associated with 3d. The pink module (113 transcripts) was highly associated with 5d (Supplementary Table S8).

**FIGURE 3:**
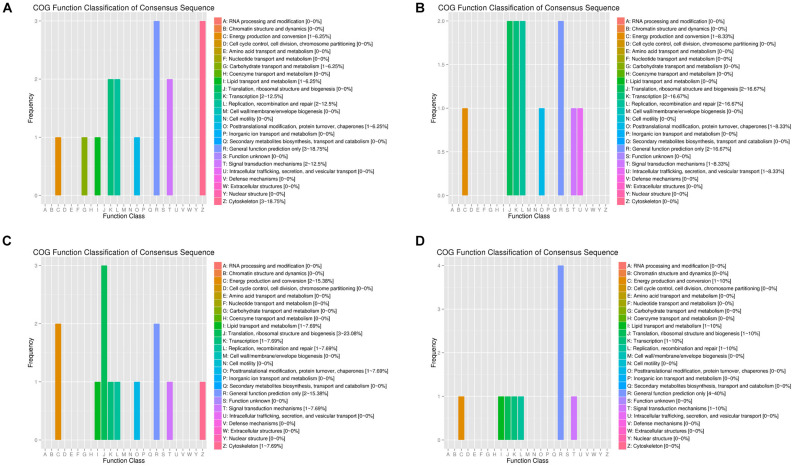
COG function classification of the module hub genes. **(A)** Turquoise module; **(B)** blue module; **(C)** green module; **(D)** pink module.

**FIGURE 4:**
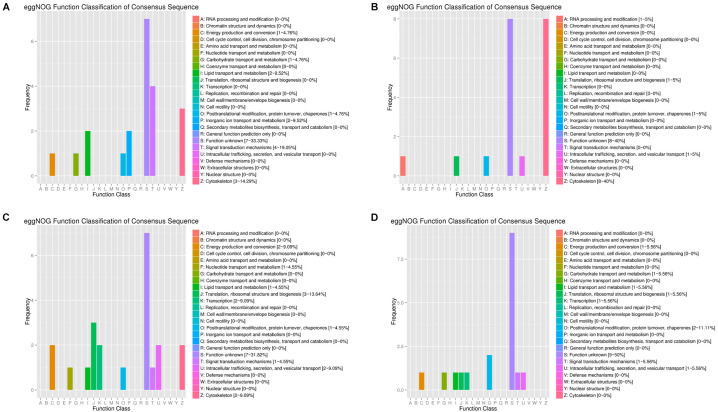
eggNOG function classification of the module hub genes. **(A)** Turquoise module; **(B)** blue module; **(C)** green module; **(D)** pink module.

**FIGURE 5:**
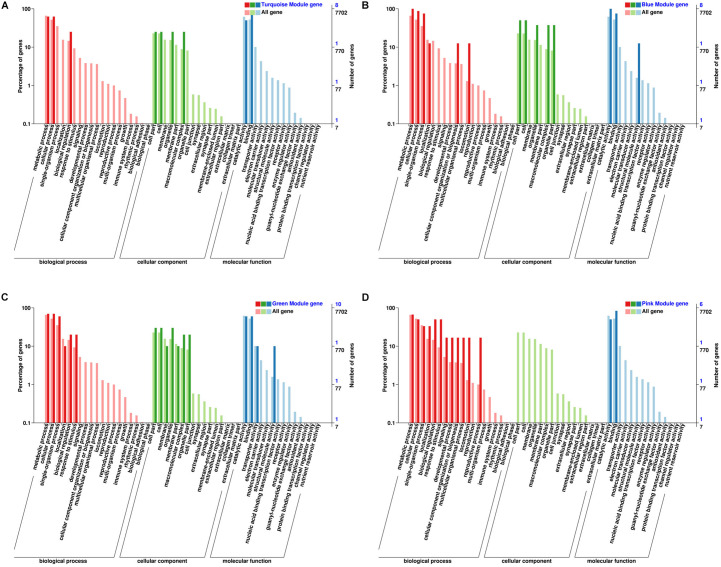
Classification and statistics of GO annotation with the module hub genes. **(A)** Turquoise module; **(B)** blue module; **(C)** green module; **(D)** pink module.

By arranging weight values in descending order, highly connected transcripts in the four specific modules were defined as hub genes and functional analysis was performed on these hub genes (Supplementary Table S9). It can be seen from the various database annotation that there were a great number of hub genes with unknown function or predictive function in each module (Figures 3, 4). It indicated that many unknown transcripts may play important roles during planarian regeneration, but their function remained to be explored. The most molecular functions that hub genes participated in each module are catalytic activity and binding (Figure 5), indicating that these two physiological processes are essential in the homeostatic maintenance and regeneration process.

**FIGURE 6:**
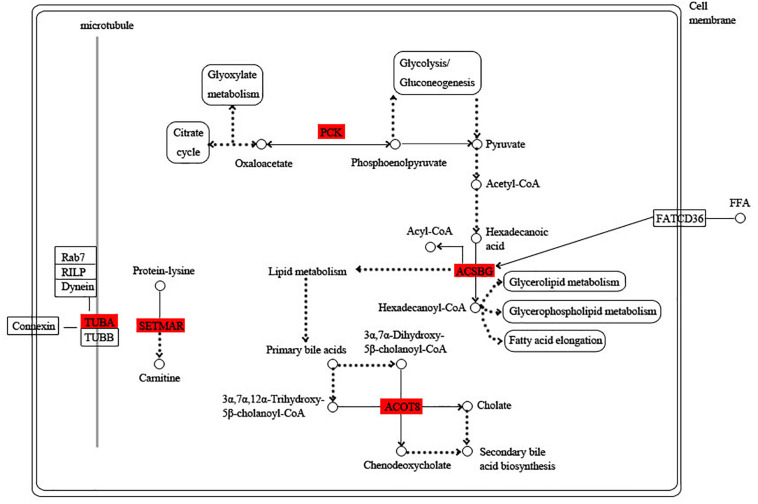
Functional relationship network of key genes in turquoise module. Rectangle represents protein, rounded rectangle represents signal pathway, circle represents metabolite, line represents direct action, dot line represents indirect action. Arrow represents direction of action, T-line represents inhibition.

Among these hub genes, 8, 6, 10, and 7 hub genes were annotated into the KEGG pathway in turquoise, blue, green, and pink modules, respectively (Supplementary Table S9). Based on the functional annotation and the involved KEGG pathway of these hub genes, the gene networks of different modules were integrated, in which the proteins highlighted in red were encoded by the hub genes. In the turquoise module, energy circulation and metabolism are the main physiological activities. For example, citrate cycle, pyruvate metabolism, glycolysis and fatty acid metabolism provide energy for the growth and homeostatic maintenance of the planarians. In addition, the regulation of microtubule is also essential for the transport of substances and cell division (Figure 6).

**FIGURE 7:**
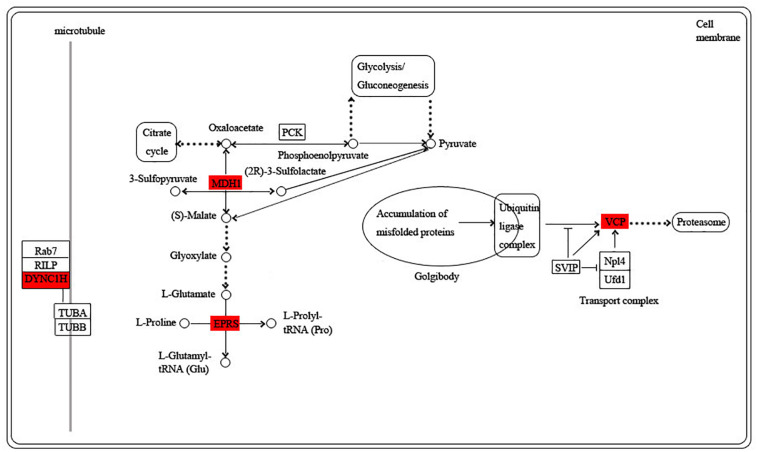
Functional relationship network of key genes in blue module.

**FIGURE 8:**
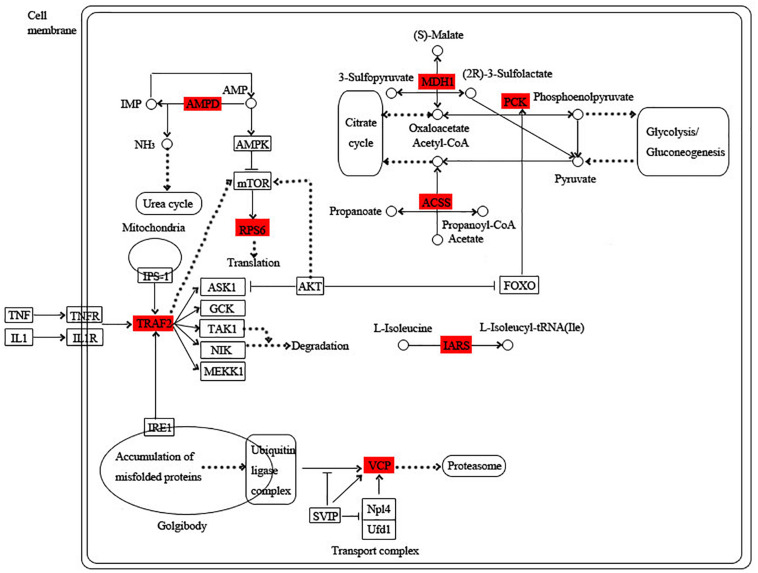
Functional relationship network of key genes in green module.

**FIGURE 9:**
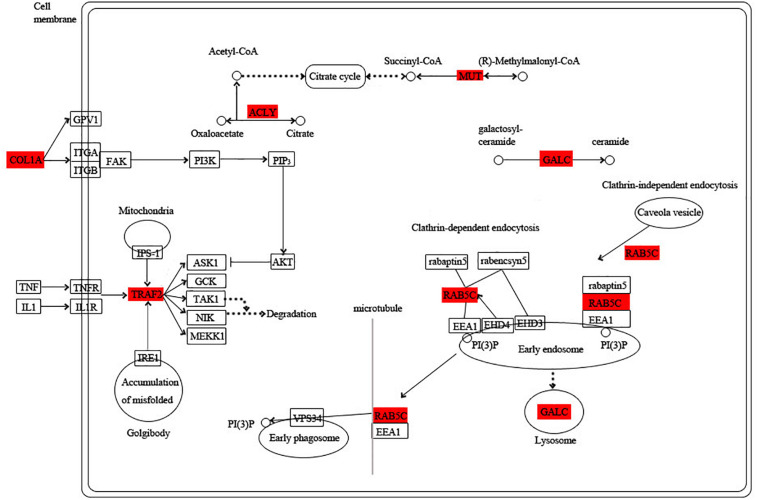
Functional relationship network of key genes in pink module.

The blue module is specific to the 1st day of regeneration. Besides the energy-generating activities including citrate cycle, pyruvate metabolism, and glycolysis, the physiological activities of hub genes also involve dynein, translation, and degradation of misfolded proteins (Figure 7). In the green module specific to the 3rd day of regeneration, citrate cycle, pyruvate metabolism, glycolysis, fatty acid metabolism, AMP hydrolysis co-regulate energy production, and circulation. And translation and degradation of misfolded proteins remain the main physiological activities. In addition, TRAF2 is a key signaling protein involved in the inflammatory and apoptosis (Figure 8). In the pink module, the physiological activities that regulate energy cycling are only the citrate cycle and fatty acid metabolism. In addition, COL1A is involved in intercellular interactions, and TRAF2 is involved in apoptosis. Endocytosis has also become a major physiological activity (Figure 9).

### Hypothesized Regeneration Pattern in *D. japonica*

**FIGURE 10:**
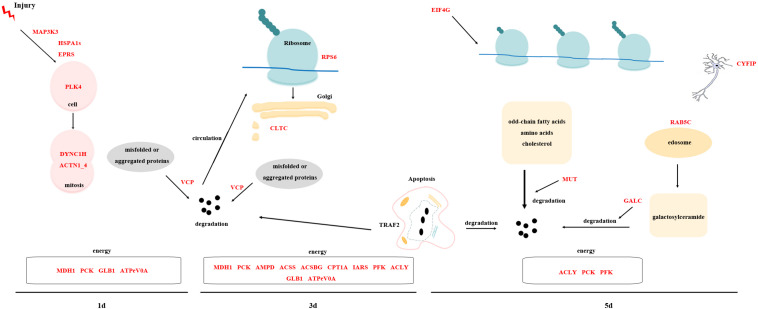
Cartoon displaying the hypothesis on regeneration pattern in *D. japonica*.

By analyzing the overall gene expression profiles of regeneration, 63 transcripts were screened out as potential key transcripts for regeneration (Tables 3–5 and Supplementary Table S9). Many of these transcripts are predicted to be the same protein. Based on their predicted proteins, we proposed the following regeneration pattern in *D. japonica* (Figure 10). On the 1st day of regeneration, the damage signal was transmitted to the cell via the MAPK signal pathway. The process of signal transmission may involve bifunctional glutamyl/prolyl-tRNA synthetase (EPRS) and HSPA1s. After that, PLK4 was significantly up-regulated, suggesting that the cell division activity increased. Dynein heavy chain (DYNC1H) and ACTN1_4 assisted in cell division. The misfolded protein produced during this period was degraded by transitional endoplasmic reticulum ATPase (VCP). Meanwhile, malate dehydrogenase (MDH1), PCK, GLB1, and ATPeV0A were involved in providing the energy needed for cell division. On the 3rd day of regeneration, small subunit ribosomal protein S6e (RPS6) was involved in protein synthesis, TRAF2 was involved in cell apoptosis and inflammatory response. The expression level of CLTC was significantly increased, indicating transport activity increased. The misfolded protein produced during this period was degraded by VCP. At the same time, MDH1, PCK, AMP deaminase (AMPD), acetyl-CoA synthetase (ACSS), ACSBG, CPT1A, isoleucyl-tRNA synthetase (IARS), PFK, ACLY, GLB1, and ATPeV0A were involved in providing the energy required for the above physiological activities. On the 5th day of regeneration, the expression levels of EIF4G, CYFIP, and TRAF2 were significantly increased, and it is speculated that translation, neural development and apoptosis activity increased. In addition, Ras-related protein (RAB5C) participated in endocytosis and may assist the methylmalonyl-CoA mutase (MUT) and Galactosylceramidase (GALC) in the material degradation process. ACLY, PCK, PFK were involved in providing the energy required for the above physiological activities.

## Discussion

Regeneration is a complex process and is the result of comprehensive regulation of various physiological processes. In this study, the full-length transcriptome sequencing with different regeneration time points was performed on *D. japonica* to obtain a more accurate and comprehensive transcript sequences. In view of the high error rate of the third-generation sequencing and the lack of reliable reference genome in *D. japonica*, a set of bioinformatics process for the integration and analysis of the second-generation and third-generation transcriptome sequencing data was established to accurately realize the error correction. The results provided more comprehensive information on genes involved in the regeneration. This study also showed that WGCNA is particularly useful in identifying sample-specific modules and hub genes. WGCNA provides a unique way to help classify large amounts of data, summarize useful information and screen out key genes.

In this study, we screened key transcripts and important signaling pathways from two approaches. The first method is to screen the DETs and their involved pathways. The second method is to screen the hub genes by WGCNA. Through the combination of these two methods, we conducted preliminary screening of important transcripts in the regeneration process, which will provide a basis for further in-depth research.

Among the predicted proteins of selected transcripts, MDH1 participates in the citrate cycle, catalyzing the dehydrogenation of L-malic acid and interconversion with oxaloacetate (Eprintsev et al., 2018). ACLY, which catalyzes the formation of acetyl-CoA and oxaloacetate from citrate and CoA, is a positive regulator in glycolysis (Watson et al., 1969). ATPeV0A is a major functional domain of the V-type H+-transporting ATPase and plays an important role in proton transport (Zhang et al., 1994). PCK catalyzes the reversible conversion of oxaloacetate and phosphoenolpyruvate and is a key enzyme in glycolysis and gluconeogenesis (Matte et al., 1997). PFK, the main rate-limiting enzyme in the glycolysis, catalyzes the production of fructose-1, 6-bisphosphate from fructose 6-phosphate (Schöneberg et al., 2013). These proteins participated in the energy cycle by participating in the citrate cycle and glycolysis. GLB1 is an enzyme that hydrolyzes lactose to glucose and galactose (Juers et al., 2001). HSPA1s is a cytoplasmic chaperone that promotes protein folding, degradation, complex assembly, and translocation (Mayer, 2010). VCP is an ATPase with multiple cellular functions. Its primarily function is target misfolded or aggregated proteins to degradation pathways, including the endoplasmic reticulum-associated degradation (ERAD) pathway (Wolf and Stolz, 2012). TNF receptor-associated factor 2 (TRAF2) is a member of the TRAF superfamily that acts as a key signal transduction protein and is involved in inflammatory and apoptosis (Inoue et al., 2000). The above proteins were involved in multiple regenerate stages. Therefore, glycolysis is a very important metabolic activity in the regeneration process, together with the citrate cycle to provide sufficient energy for other physiological activities. In addition, protein degradation and apoptosis also promote the circulation of materials required for regeneration.

Among the key proteins in the 1st day of regeneration, MAP3K3 is a member of the mitogen-activated protein kinase family. MAPKs signal pathway exists in most cells types, plays a crucial role in the process of transferring extracellular signals into cells and then causes cell biological responses (Craig et al., 2008). Therefore, MAP3K3 may be responsible for transmitting damage signals on the 1st day of regeneration. Heat shock protein and MAPK pathway mediate early response of regeneration, which are consistent with the conclusion of this transcriptome data (Yuan et al., 2018). PLK4 plays a key role in centriole replication, and the loss of its function disrupts mitosis (Habedanck et al., 2005). Up-regulation of PLK4 indicates an increase in cell proliferation activity on the 1st day of regeneration. DYNC1H can move along microtubule, participates in the transport of substances in the cytoplasm and assists in mitosis (Porter, 1996; Vale, 2003; Pfister et al., 2006). ACTN1_4, an important actin cross-linking protein in the cytoskeleton, plays an important role in regulating cell adhesion, cell shape and movement through synergy with its related proteins (Hynes, 1992). DYNC1H and ACTN1_4 may assist in cell division. EPRS can catalyze the binding of glutamate and proline to the corresponding tRNA, identify tRNA in the first step of protein biosynthesis to ensure the high accuracy of mRNA translation. In addition, there was a literature indicating that EPRS release from the aminoacyl tRNA multisynthetase complex, which is required for execution of non-canonical functions beyond protein synthesis (Arif et al., 2009). For example, phospho-EPRS binds fatty acid transport protein 1 (FATP1), inducing its translocation to the plasma membrane and the uptake of long-chain fatty acid (Arif et al., 2017). EPRS is also involved in the regulation of inflammatory responses (Lee et al., 2016). Proliferation is the main activity on the 1st day of regeneration. The cells with proliferation ability in *D. japonica* were named neoblasts. Neoblast proliferates rapidly after injury and there will be two peaks of proliferation: 6 and 48 h after cutting, respectively (Wenemoser and Reddien, 2010). The 1st day of regeneration is between the two time points, so proliferation may remain the main physiological activity.

On the 3rd day of regeneration, most transcripts were involved in the production of energy, such as AMPD, ACSS, ACSBG, and CPT1A. ACSBG participates in fatty acid (FA) metabolism that converts free long-chain FA to acyl-CoA (Mashek et al., 2007). CPT1A regulates the transport of fatty acids on the mitochondrial membrane in invertebrates and plays a major role in the fatty acid oxidation pathway (Bartlett and Eaton, 2004). It may be necessary to provide more energy on the 3rd day to meet the needs of regeneration. At the same time, translation process increased with RPS6 and IARS on the 3rd day of regeneration. CLTC involved in the transport of substances was significantly up-regulated at 3d. It relates to coating membranes that are endocytosed from plasma membranes and those that move between the trans-Golgi network and endosomes (Kirchhausen, 2000).

Among the key proteins in the 5th day of regeneration, there are some proteins involved in the degradation of substances. For example, MUT is required for the degradation of odd-chain fatty acids, amino acids (such as valine, isoleucine, methionine and threonine) and cholesterol (Martínez et al., 2005). GALC is the lysosomal β-galactosidase that is responsible for the hydrolysis of galactosylceramide. The accumulation of galactosylceramide and its deacylation cause neurological diseases (Deane et al., 2011). In addition, RAB5C is involved in the regulation of early endosomal fusion during endocytosis (Li and Stahl, 1993). Collagen (COL1A) is deposited in the extracellular matrix and helps to regulate the mechanical properties and shape of the tissues. It interacts with cells through several receptor families to regulate cell proliferation, migration, and differentiation (Humphries et al., 2006; Heino, 2007; Leitinger and Hohenester, 2007; Heino et al., 2009). These two proteins may assist in the degradation of substances. Meanwhile, EIF4G, which plays a major role in the initiation of protein synthesis (LeFebvre et al., 2006), is significantly up-regulated. In summary, on the 5th day of regeneration, activities such as translation, transport and degradation of proteins are active to meet the needs of various physiological activities in the regeneration process. In addition, CYFIP which is involved in the development and maintenance of neuronal structures showed increased expression, indicating that the neurodevelopment of the planarians appears to increase on the 5th day (Han et al., 2015).

## Conclusion

In general, energy is necessary for planarian regeneration. While at different time points of regeneration, the physiological activities in the worms are emphasized. On the 1st day of regeneration, mitosis is carried out in the worms. On the 3rd day of regeneration, more energy metabolism activities are carried out. Translation, degradation of protein and neural development activities increase on the 5th day of regeneration. We screened out the above important transcripts involved in regeneration to conduct further exploration in the next study. In addition, there are a large number of transcripts in the planarian transcriptome with unknown function, which may also be involved in important physiological activities to complete regeneration. On the whole, this study provides a comprehensive data foundation for further excavation of planarians regeneration.

## Data Availability Statement

The data set supporting the results of this article are available in the Sequence Read Archive (SRA) of NCBI (accession no. PRJNA627589).

## Ethics Statement

This study does not involve endangered or protected species, and the collection of specimen is approved by the Forestry Department of Wild Animal Protection, Henan Province, China.

## Author Contributions

YY analyzed the transcriptome data and drafted the manuscript. PW performed qRT-PCR. BJ uploaded the transcriptome data. ZD and GC conceived the study and participated in its design. DL had been involved in revising the manuscript. All authors have read and approved the manuscript.

## Conflict of Interest

The authors declare that the research was conducted in the absence of any commercial or financial relationships that could be construed as a potential conflict of interest.

## Abbreviations

ACLY: ATP citrate (pro-S)-lyase
ACSBG: Long-chain-fatty-acid–CoA ligase
ACTN1_4: actinin alpha 1/4
ADCY9: adenylate cyclase 9
ATPeV0A: V-type H+-transporting ATPase subunit a
CLTC: clathrin heavy chain
CPT1A: carnitine O-palmitoyltransferase 1
CYFIP: cytoplasmic FMR1 interacting protein
DET: differentially expressed transcript
DPYS: dihydropyrimidinase
EIF4G: translation initiation factor 4G
FPKM: fragments per kilobase of transcript per million mapped reads
GLB1: beta-galactosidase
HSPA1s: heat shock 70 kDa protein 1/2/6/8
MAP3K3: mitogen-activated protein kinase kinase kinase 3
MDH1: malate dehydrogenase
PCK: phosphoenolpyruvate carboxykinase
PFK: 6-phosphofructokinase 1
PK: pyruvate kinase
PLK4: polo-like kinase 4
ROI: reads of insert
SMRT: single-molecule real-time
TRAF2: TNF receptor-associated factor 2
VCP: transitional endoplasmic reticulum ATPase
WGCNA: weighted gene co-expression network analysis.
