# Supplementary material for: Screening of Potential Key Transcripts Involved in Planarian Regeneration and Analysis of Its Regeneration Patterns by PacBio Long-Read Sequencing
Source: Front Genet. 2020 Jun 16;11:580. doi: 10.3389/fgene.2020.00580 (PMC7308552; doi:10.3389/fgene.2020.00580)
Supplement: Supplementary file 1 [file Table_1.DOC]

**Table. S1 All primers used for this study.**

| **Primers** | **Sequence(5'-3')** |
| --- | --- |
| *DjEf2*/RT/F | AAGACGATGAAGTTGCTATTGC |
| *DjEf2*/RT/R | AACTCCGACAAGACCACAGAT |
| F01.PB35778/RT/F | TCCTGCAAGAACATGCTTACATC |
| F01.PB35778/RT/R | CAAATTGAGACGCTTTATTGCTG |
| F01.PB3115/RT/F | GGTCCCGTTTCCTCGTATTC |
| F01.PB3115/RT/R | CAATCATTAGCAAGTTCAGGCAC |
| F01.PB13715/RT/F | ATCAAAAGGCACAAACGCTG |
| F01.PB13715/RT/R | GTGGCTCCTACAAATACACCG |
| F01.PB27496/RT/F | GATGGAGATGTTGCCACTTATG |
| F01.PB27496/RT/R | CTTGTGACTTAACCTCTGGCG |
| F01.PB13910/RT/F | CCTGGGAGGATATTGGTGGTC |
| F01.PB13910/RT/R | CGCCTTTGCTTGGAGTCATAC |
| F01.PB11703/RT/F | GCATCTGCCCCTATTGCTGAG |
| F01.PB11703/RT/R | CGATTTAGGAAGTCCCTGTCCC |
