# Supplementary material for: Screening of Potential Key Transcripts Involved in Planarian Regeneration and Analysis of Its Regeneration Patterns by PacBio Long-Read Sequencing
Source: Front Genet. 2020 Jun 16;11:580. doi: 10.3389/fgene.2020.00580 (PMC7308552; doi:10.3389/fgene.2020.00580)
Supplement: Supplementary file 3 [file Table_3.DOC]

Table. S3 Numbers of differentially expressed transcripts.

| **DETs set** | **DETs in total** | **Annotated DETs** | **DETs annotated in KEGG** | **Number of pathways DETs involved in** |
| --- | --- | --- | --- | --- |
| T01_T02_T03_vsT04_T05_T06 | 5931 | 5066 | 2558 | 251 |
| T01_T02_T03_vsT07_T08_T09 | 5115 | 4432 | 2182 | 238 |
| T01_T02_T03_vsT10_T11_T12 | 4669 | 4057 | 2058 | 228 |
