# Supplementary figures and images for: Screening of Potential Key Transcripts Involved in Planarian Regeneration and Analysis of Its Regeneration Patterns by PacBio Long-Read Sequencing
Source: Front Genet. 2020 Jun 16;11:580. doi: 10.3389/fgene.2020.00580 (PMC7308552; doi:10.3389/fgene.2020.00580)

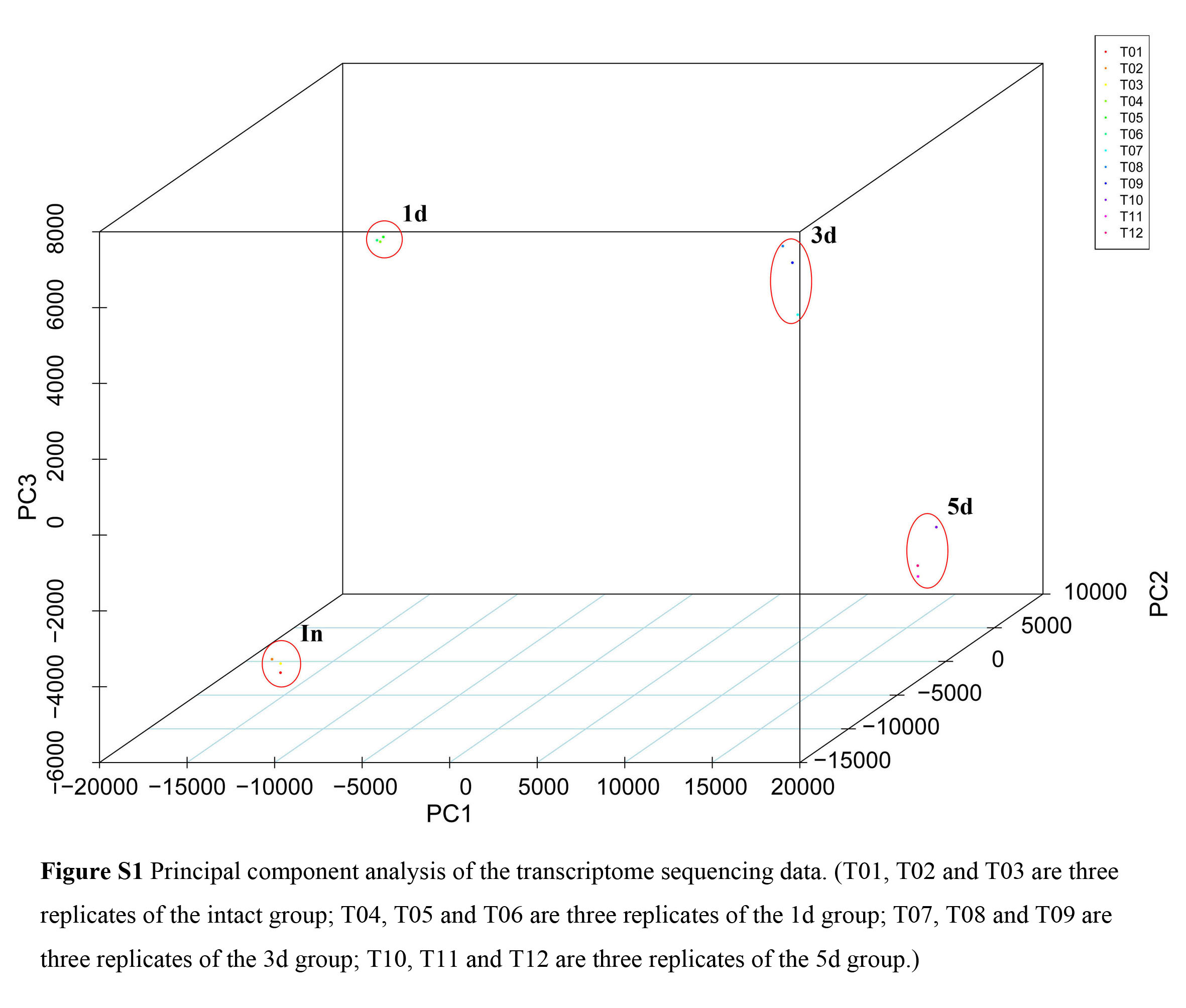

Supplement: Supplementary file 6 [file Image_1.jpg]

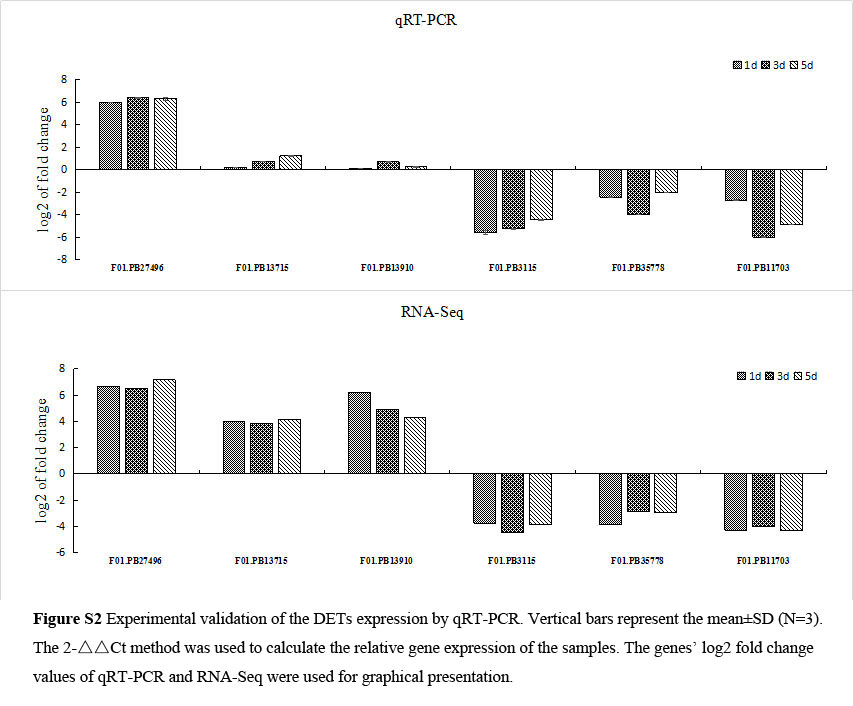

Supplement: Supplementary file 7 [file Image_2.jpg]
